# Understanding Patient Experience with Outpatient Cancer Rehabilitation Care

**DOI:** 10.3390/healthcare11030348

**Published:** 2023-01-25

**Authors:** Kelley C. Wood, Jessica J. Bertram, Tiffany D. Kendig, Mackenzi Pergolotti

**Affiliations:** 1 ReVital Cancer Rehabilitation, Select Medical, Mechanicsburg, PA 17050, USA; 2 Outpatient Division, Baylor Scott and White Institute for Rehabilitation, Dallas, TX 76132, USA; 3 Department of Occupational Therapy, Colorado State University, Fort Collins, CO 80523, USA; 4 Department of Occupational Science and Occupational Therapy, University of North Carolina, Chapel Hill, NC 27599, USA

**Keywords:** patient acceptance of healthcare, patient satisfaction, rehabilitation, quality of healthcare, cancer survivors

## Abstract

Background: Understanding patient experience is key to optimize access and quality of outpatient cancer rehabilitation (physical or occupational therapy, PT/OT) services. Methods: We performed a retrospective mixed-method analysis of rehabilitation medical record data to better understand patient experience and aspects of care that influenced experience. From the medical record, we extracted case characteristics, patient experience data (Net Promoter Survey^®^, NPS) and patient-reported outcome (PRO) data. We categorized cases as ‘promoters’ (i.e., highly likely to recommend rehabilitation) or ‘detractors’, then calculated NPS score (−100 [worst] to 100 [best]). We identified key themes from NPS free-text comments using inductive content analysis, then used Pearson [*r*] or Spearman [*ρ*] correlation to explore relationships between NPS, characteristics, and PRO improvement. Results: Patients (n = 383) were 60.51 ± 12.02 years old, predominantly women with breast cancer (69.2%), and attended 14.23 ± 12.37 visits. Most were ‘promoters’ (92%); NPS score was 91.4. Patients described two experiences (themes) that influenced their likelihood to recommend rehabilitation: (1) feeling comfortable with the process and (2) observable improvement in health/functioning, and described attributes of clinic staff, environment and clinical care that influenced themes. Likelihood to recommend rehabilitation was associated with achieving the minimal clinical important difference on a PRO (*ρ* = 0.21, *p* < 0.001) and cancer type (*ρ* = 0.10, *p* < 0.001). Conclusion: Patients who received specialized cancer PT/OT were highly likely to recommend rehabilitation. Feeling comfortable with the rehabilitation process and making observable improvements in health and/or functioning influenced likelihood to recommend. Rehabilitation providers should leverage the findings of this study optimize access to and quality of cancer rehab services.

## 1. Introduction

The degree to which health care services meet the needs of those who use them, or patient experience, is an important dimension of health care quality [1,2]. Months to years following treatment, 60 to 90% of adults living with and beyond cancer (i.e., cancer survivors) report unmet physical health (e.g., chronic pain, lymphedema, neuropathy, gait and balance disturbance) and functioning needs (e.g., self-care, home-care, return to work) but as a few as 10% utilize rehabilitation services (e.g., physical and occupational therapy, PT/OT) [3,4,5,6]. Systematic reviews demonstrate the efficacy of cancer-specialized PT/OT interventions to ameliorate physical health and functioning [7,8], and analysis of real-world outpatient services show patients find them to be acceptable and experience improvement in physical and functional health [9,10,11]. In addition, oncology clinical practice guidelines recommend referral to these services [12]. However, oncology providers and survivors cite low awareness of cancer rehabilitation services [13,14,15,16]. Oncology providers report little training and poor knowledge of patients functional needs, including when and how patients may benefit from referral to rehabilitation services [13,14,17]. Furthermore, qualitative studies show functional decline is a significant source of distress after cancer surgery [18], and that participation in rehabilitation programs can serve as a “stepping stone to normality” by helping them to improve strength, manage treatment of late/lasting effects and regain functional independence [19,20].

Increased use of PT/OT services may be key to address a cancer survivor’s physical health and functional needs, but research evidence is needed to understand the survivor’s perspective of these programs [14]. Current research provides limited insight into the experiences of those who participate in outpatient cancer rehabilitation services [14,16]. Understanding the degree to which cancer rehabilitation services meet survivors’ needs is critical to help oncology providers and patients see the value of these services in oncology care and patients’ lives, and ultimately to enhance referrals to and use of rehabilitation services [14]. In addition, for rehabilitation providers, better understanding of the aspects of care that matter the most to patients could be key to proactively address patients’ needs and consistently deliver high-quality rehabilitation care. To enhance understanding of patient experience, researchers call for the collection and analysis of patient-reported experience and qualitative data from real-world outpatient cancer rehabilitation services [14,21,22]. To better understand patient experience and aspects of care that influenced their experience, we performed a mixed methods analysis of data collected from participants in a national outpatient cancer rehabilitation program.

## 2. Materials and Methods

We report study methods following the Professional Society for Health Economics and Outcomes Research (ISPOR) checklist for retrospective studies [23].

### 2.1. Study Population

Data was extracted from the institution’s medical record and provided to the researchers as a partially de-identified data set. Cases met the following inclusion criteria: (1) attended at least two community-based outpatient cancer rehabilitation appointments in 2019 delivered by a physical (PT) and/or occupational therapist (OT), (2) primary cancer type identifiable by International Classification of Diseases (ICD)-10 code, (3) completed NPS, and (4) completed at least one patient reported outcome (PRO) measure at initial evaluation and within seven days of discharge.

### 2.2. Net Promoter Survey^®^

The Institute of Healthcare Improvement recognizes a patient’s likelihood to recommend healthcare services as a proxy for patient experience and as an important aspect of healthcare quality [1]. The Net Promoter Survey^®^ (NPS) is a commonly used two-item patient-reported experience measure in healthcare [24]. NPS item #1 asks: “*How likely are you to recommend (this facility) to family/friends*?”; patients’ rate on a 11-point Likert scale (0 [not at all likely] to 10 [extremely likely]). NPS item #2 asks: “*What is the most important reason for your score?*”; patients’ provide a free-text response. We scored item #1 at an individual and aggregate level based on NPS scoring guidelines [21]. At an individual level, we categorized those who respond 9 to 10 as “promoters”, those who respond 7–8 as “passives” and those who responded 6 and below “detractors”. At an aggregate level, we calculated the NPS score by subtracting the proportion of detractors from the proportion of promoters (e.g., % promoters–% detractors). Possible range in NPS score is −100 to 100; lower score indicates poorer experience [21]. Across health care and business, an NPS score greater than or equal to 0 is considered acceptable; in outpatient rehabilitation, the suggested benchmark for NPS score is 84 [25].

The rehabilitation institution administered NPS via email to rehabilitation patients following established guidelines and industry standards [25,26]. NPS was emailed up to three times during therapy—after initial evaluation, six weeks after initial evaluation, and following discharge. For this study, we extracted NPS data available closest to discharge date for all eligible cases.

### 2.3. Independent Variables

Individual and rehabilitation characteristics available for extraction included: age (years), sex (male or female), cancer type (grouped by type based on ICD-codes), United States geographical region (based on U.S. census groupings), rehabilitation discipline (PT or OT), number of visits and weeks per episode. For each case, we examined patient pre- and post-rehabilitation scores on PRO selected by the treating therapist, then coded whether the patient achieved the measure-specific validated minimal clinical important difference (MCID). MCID indicates the “smallest improvement considered worthwhile by a patient” [27]. The following outcome measures were used: the Quick Disabilities of the Arm, Shoulder, and Hand (MCID = 15.91 points [28]), the Lower Extremity Functional Scale (MCID = 9 points [29]), and the Modified Low Back Pain Disability Questionnaire (MCID = 7.5 points) [30].

### 2.4. Statistical Analysis

We performed parallel mixed method analysis using NPS ratings (0–10), NPS comments, and independent variables [31]. We examined NPS ratings descriptively, then used Pearson (*r*) or Spearman rank correlation (*ρ*) to examine relationships between NPS rating and independent variables (age, sex, cancer type, attendance, duration, and achievement of the MCID). Significance level for correlation analyses was *p* < 0.05.

### 2.5. Qualitative Analysis

To better understand patients’ perspectives, we performed inductive content analysis of NPS comments following established guidelines [32,33]. Two independent reviewers (KW and JB) appraised participant responses to identify key content described by participants. Reviewers grouped key content into categories to establish an initial coding frame, then refined codes over two rounds of review until no new content emerged. Comments that did not include a clear subject (e.g., staff, clinical care, environment) and descriptor were excluded at this stage, resulting in 190 comments (78%) eligible for coding and analysis. For example, one participant wrote, “good care”; without a subject, this comment was excluded from analysis because it is not clear whether “good care” was related to staff or treatment. The final coding list included three categories and six codes (two for each category) and was used by each reviewer to independently code each eligible comment. A third reviewer (MP) was available for final decision when consensus was not reached between reviewers. For comments that included content from more than one category, reviewers coded all unique content [34]. Coding inter-rater agreement was 100%. Once coding agreement was achieved, reviewers identified patterns (themes) among the categories to better understand the essence of the patient experience and identified quotes that accurately depicted themes [35].

## 3. Results

Patient cases that met inclusion criteria (n = 383) had a mean age of 60.51 ± 12.02 years old, were mostly female (84.3%), and had the following cancer types: breast (69.2%), heme or lymphoid (7.6%), musculoskeletal (e.g., bone, soft or connective tissue; 6.3%), or other (17.0%). They attended PT (n = 341, 89.0%) or OT (n = 42, 11.0%) in 176 clinic locations across the U.S. Regardless of rehabilitation discipline, they attended an average of 14.23 ± 12.37 sessions over 11.17 ± 11.25 weeks. All characteristics are reported in Table 1.

### 3.1. Quantitative Findings

NPS score was 91.4, 92.2% were ‘promoters’ (n = 353), 7.0% were ‘passives’ (n = 27), and 0.8% were ‘detractors’ (n = 3). A weak but significant correlation was observed between NPS rating and achieving the MCID on a PRO (*ρ* = 0.16, *p* = 0.002). No significant correlations were observed between NPS rating and the following patient or rehabilitation characteristics: age (*r* = −0.03, *p* = 0.15), sex [*ρ* = −0.06, *p* = 0.24], number of visits [*r* = 0.06, *p* = 0.26] or weeks attended [*r* = −0.02, *p* = 0.72]). A small but significant correlation was observed between NPS score and cancer type (*ρ* = 0.10, *p* ≤ 0.001); lowest average NPS ratings were provided by individuals with endocrine/neuroendocrine (e.g., thyroid, pituitary gland, or other nervous system; n = 4, median = 9.0, IQR = 8.25–9.75) and gastrointestinal (n = 6, median = 10.0, IQR = 7.7–10.0) cancer types.

### 3.2. Qualitative Findings

**Figure 1:**
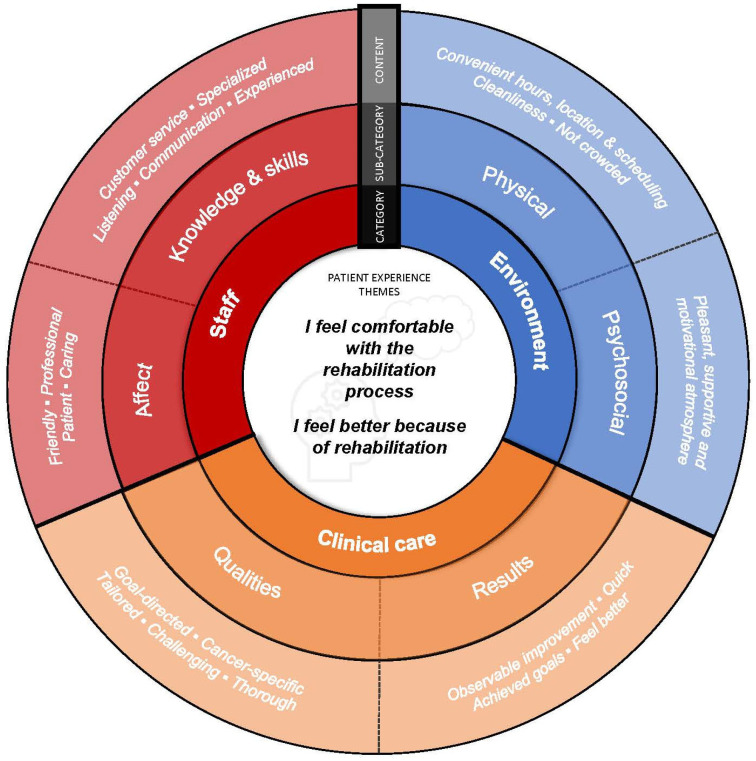
Aspects of patient experience associated with recommending cancer rehabilitation services.

Sixty-four percent of respondents provided free text comments (n = 245). Of those, 95.5% were ‘promoters’ (n = 234), 4.5% were ‘passives’ (n = 11), and none were ‘detractors’. We identified two distinct themes from free-text NPS comments—(1) patients felt comfortable with the rehabilitation process and (2) patients felt better because of rehabilitation. Within each theme, three categories influenced patient’s NPS rating: staff (sub-categories: affect and knowledge/skills), clinical care (sub-categories: qualities and results), and the clinic environment (sub-categories: physical and psychosocial). Each category is presented in Figure 1 with corresponding sub-categories and key content identified from patient quotes. Each theme could positively (i.e., enhance) or negatively influence (i.e., diminish) patient experience; however, less than 3% of all comments included a ‘negative’ descriptor. As such, we present each theme below using the ‘positive’ perspective.

#### 3.2.1. Theme 1: I Felt Comfortable with the Rehabilitation Process

“*The total experience is important to me on my oncology journey … the consistently kind and caring front office staff to the highly skilled and compassionate care from the professional therapist are why I trust and recommend this facility.*”

(ID#208)

From scheduling their first appointment to working with their therapist, patients described that they felt “comfortable” with the rehabilitation process, citing positive interactions with staff, qualities of their care, and aspects of the environment. Several commented that clinic locations were “convenient”, and scheduling was “accommodating” to their busy work and/or cancer treatment schedules. Interactions with office staff were critical to help patients feel comfortable. One commented, “*the ladies in the reception were always cheerful and made me feel welcome*” (ID#38). Many favorably described “customer service” provided by office staff and office management—for example, making sure appointments began on time to minimize waiting room crowding and patient wait times. Overall, the common sentiment was that office staff were “*professional and competent and make the office environment very pleasant*” (ID#122).

During therapy appointments, the therapist affect, knowledge, and skills helped patients to feel at ease and have clear expectations of the rehabilitation process. For example, one patient commented: “*[My therapist] was able to make me feel comfortable with the whole process, she was able to ease some of my fears and explain what I can expect throughout the process of cancer rehab*” (ID#147). One patient commented that, although they were “*very nervous coming*”, they soon became “*extremely comfortable*” because their therapist was “*extremely knowledgeable, compassionate and very sensitive to my therapy and care*” (ID#118). These initial positive experiences often manifested into long-term trust with their therapist. For example, one patient commented: “*My therapist is professional but friendly and caring, she is very knowledgeable, and I have complete confidence in her direction of treatment for me*” (ID#117). Patients who worked with more than one therapist described a similar experience with each therapist they encountered, for example: *“The therapists that I have worked with there are very knowledgeable about all aspects of working with the clients … everyone is friendly and cares about their clients … they go beyond considering what is best for each person*” (ID#207).

Many patients commented that because they felt comfortable with their therapist and trusted their plan of care, it “*makes the time go quickly (and) I don’t mind going back to see [my therapist]*” (ID#76). Ultimately, patients described positive experiences with office staff, in the clinic environment, and support from their therapist helped them to leave therapy sessions feeling hopeful: “*The physical therapist is very friendly and makes me feel very comfortable she also explains clearly what she is doing I walk away feeling very hopeful.*” (ID#180).

#### 3.2.2. Theme 2: I Feel Better

“*The whole team from the administrative staff to therapists are supportive and encouraging … they assess where you are at what you need and they challenge you to go beyond what you think you can do. There is a marked difference in what I can do now and feel*”

(ID#210)

During rehabilitation appointments, patients described their therapist’s affect, knowledge and skills as critical to helping them to achieve their rehabilitation goals. For example, one participant commented: “*The therapists are patient, listen to what the clients need in order to recover … they encourage but gently push the client toward their goals … I feel like my mobility has improved*” (ID#204). Another stated, “*My therapist [name] is wonderful we have made progress because she listens to my concerns and tweaks my program to maximize my improvement she has helped me so much the facility is clean everyone there is very professional*” (ID#113). Therapists were focused on patients’ needs and specifically tailored each session to help them make observable progress towards their personal goals. One participant stated:

“*The goals that I set for myself at the beginning of therapy were all met by the end of my sessions … all of the exercises and methodologies that were used were to help me reach all of my goals successfully … the staff was professional and knowledgeable*”

(ID#170)

Overall, patients described noticeable improvements in their strength, health and/or well-being as well as gratitude to their therapist and/or referring physician, for example: “*I gave the facility and [my therapist] all a 10 because {my therapist] is the greatest my pain and stress level decreased from day one I am glad my physician referred me here*” (ID#67). One patient specifically attributed their high loyalty (i.e., 10/10 NPS rating) to the therapists who helped them to see quick and meaningful improvements in their health: “*The personal attention and care of the staff are the reason for my score. I saw so much improvement in a short time due to the custom program created for me*” (ID#179). In addition to observable improvement, many recognized their therapist for teaching them skills to help them maintain these improvements outside of therapy visits, for example “*I appreciate her desire to educate me on how best to take care of myself and help me to do my exercises correctly*” (ID#185).

Patients also expressed gratitude to the institution for offering a *“program that is specifically targeted to treat cancer patients…”* (ID#164). Many described how their therapist helped them recover throughout cancer treatment. One patient stated: “*My therapist has helped me with recovery from multiple surgeries. I feel better, I’m happier with surgical results. In addition, she has helped me with women’s health pelvic floor issues…*” (ID#119). Another stated,

“*Very professional therapists helping to rid me of lymphedema pain while educating me on how to manage this on my own. Always on time and very pleasant. I am thankful that there is help for lymphedema as I have heard many horror stories about it.*”

(ID#240)

Overall, patients felt “*well-served*” (ID#222) by their therapist, expressing appreciation for their help during cancer treatment and skills learned to “*manage this on my own*” (ID#240).

## 4. Discussion

Advanced understanding of patient experience with cancer rehabilitation services is needed to optimize access to and quality of these services for the millions of individuals who experience cancer-related functional decline in the United States. In our previous studies, we demonstrated outpatient cancer rehabilitation services are acceptable and associated with improvement in physical health and functioning [9,10,11]. In this mixed-method study, we build on our previous work by closely examining patient experience during rehabilitation to identify aspects of care that are important to patients. These results could be proactively leveraged by rehabilitation providers to optimize patient experience and to educate cancer survivors and their providers about rehabilitation. Overall, we found over 90% of survivors who participated in rehabilitation were highly likely to recommend it to others, indicating a highly positive experience. Patients described feeling “comfortable” with the rehabilitation process and feeling “better” (i.e., improved functional health) were important aspects that positively influenced their experience. By using a mixed method approach and leveraging a national real-world patient sample (treated in over 173 clinics located across 21 states) this study provides rich but highly generalizable insight into the cancer rehabilitation patient experience. These findings should be used by researchers and accrediting or regulatory bodies (e.g., Commission on Accreditation of Rehabilitation Facilities [CARF], American College of Surgeons Commission on Cancer [CoC]) who seek to develop and recommend patient experience healthcare quality metrics for cancer rehabilitation. The themes of this study could be immediately adopted by rehabilitation providers as pillars of high-quality care delivery and by researchers to better explain the phenomenon of patient experience with cancer-specific supportive care services.

In previous studies of patient experience with rehabilitation interventions using NPS, authors found patient factors influenced likelihood to recommend rehabilitation (i.e., NPS rating), including: age [36], sex [37], clinical diagnosis (e.g., injury vs. joint replacement) [38], and perceptions of health improvement [38]. In the current study, we also found clinical diagnosis (cancer type) and making observable improvements in health or functioning (achieving the MCID on functional outcome) were associated with likelihood to recommend. For individuals with endocrine/neuroendocrine and gastrointestinal cancer types, median NPS ratings were comparatively lower than other cancer types; however, this difference may not be clinically meaningful because these groups had small sample size (n = 4–6) and median ratings were still at or above 9.0 (“promoter”).

Through evaluation of NPS free-text comments, we gained deeper insights to the aspects of care that are important to patients who received cancer rehabilitation. In these comments, we heard from patients about how interactions with staff, clinical care qualities and the clinic environment helped them to feel comfortable with the rehabilitation process and feel better—and how, ultimately, these experiences influenced their likelihood to recommend rehabilitation services to others. These data could be used by rehabilitation providers or researchers to identify “low hanging fruit” to optimize patient experience with their services via consistent delivery of high-quality PT/OT care, and to improve awareness of the value of cancer rehabilitation to oncology providers and survivors. For example, checkpoints to ensure PT/OT patients understand the rehabilitation process and have clear expectations of the co-created goals of therapy could be easily added to clinical protocols or documentation systems to promote therapists’ fidelity to these aspects in routine care. In addition, the findings of this study provide generalizable, descriptive examples of patient experience that could be used to educate oncology providers or survivors about the experience of cancer rehabilitation, potentially breaking down a previous barrier to care.

This study is the first to leverage and evaluate real-world data collected via the NPS to examine patient experience with cancer rehabilitation. In doing so, we add to the literature supporting the use of NPS as a patient experience metric in outpatient rehabilitation services and set an initial benchmark (i.e., “the bar”) for NPS for cancer rehabilitation services. This benchmark could be adopted by rehabilitation providers or accrediting organizations who seek to evaluate and/or monitor quality of services with respect to patient experience. In addition, because the NPS score in this study was higher than the accepted benchmark of 84 for general outpatient rehabilitation [25], and those reported in previous peer-reviewed studies, including general orthopedic [39] and total knee replacement populations [40,41,42] (NPS score range = 73.8 [41] to 93.0 [42]), this study provides preliminary evidence that cancer-specific services are at least “on par” with orthopedic services in terms of patient experience. Demonstrating acceptability is an important way to improve access to healthcare services. Orthopedics is a widely accepted function of rehabilitation recognized by both patients and medical providers. Therefore, ability to show cancer rehabilitation services are “on par” with orthopedic services is important to improve survivors’ and providers’ perceptions of rehabilitation.

### Limitations and Future Directions

The PRO measures available for use in this study are practical for the real-world rehabilitation context but have unique implications for research that should be considered. First, although the PRO have been validated in cancer-specific populations, the MCID values used in this study have not; therefore, may not accurately represent the level of improvement meaningful to individuals with cancer. In this study, we found less than 50% of participants achieved the MCID, but over 92% were considered promoters. The discrepancy between the proportion who achieved the MCID and promoters may be explained by low sensitivity/specificity of the establish MCID in the general cancer population, or may be evidence that patients can have a good experience despite not achieving significant improvement in function. The latter is especially relevant to the field of cancer rehabilitation because the goals of therapy can be to maintain function over the course of treatment (vs. remediate fixed impairment), therefore, no change in PRO across the course of care may be considered “successful” rehabilitation for any given patient [43]. Second, the patient experience measure used in this study (NPS) captures one domain—likelihood to recommend. We included patient comments to provide additional depth and breadth to understand experience; however, patient experience is a multifaceted construct [2]. Researchers suggest NPS may not be sufficient to capture patient experience as a stand-alone measure and should be used in tandem with more detailed surveys or validated questionnaires [21].

While this study enhances understanding of the aspects that positively influence patient experience, the data may have limited ability to understand aspects that negative influence patient experience (aside from inverse factors, e.g., poor therapist knowledge/skills) due to a low relative proportion of ‘detractors’. However, online administration of NPS is shown to be superior to in-clinic administration to capture ‘detractors’ [21]. To account for possible selection bias associated with completing the NPS (i.e., patients with a positive experience are more likely to complete), we compared characteristics and improvement on PRO between those who completed NPS (included in this study) vs. those who did not complete NPS but attended rehabilitation during the same period (excluded). In these comparisons (Supplementary Table S1), we found no significant differences in patient or rehabilitation characteristics, and no significant difference in the proportion who achieved the MCID on a PRO. Therefore, it is likely that the cases included in this study are representative of all cases. Another limitation of this study was the limited demographic and cancer treatment data available. Future research should continue to evaluate any potential influence of demographics, cancer type, treatment and timing on patient experience of rehabilitation.

## 5. Conclusions

For adults who attended outpatient cancer rehabilitation, interactions with staff, the clinical environment, and the quality of care were important aspects of patient experience that helped them feel comfortable with the rehabilitation process, feel better, and ultimately, to recommend participation in rehabilitation to others. These findings are generalizable and crucial to inform the delivery of high-quality cancer PT/OT services and to justify use of, or referrals to, these services for cancer survivors and their providers.

## Figures and Tables

**Table 1:** Patients who attended cancer physical or occupational therapy (PT/OT), n = 383.

Age (Mean ± SD)	60.51 ± 12.02
Sex (n, %)	
Female	323, 84.33%
Male	60, 15.67%
Cancer type (n, %)	
Breast	265, 69.19%
Heme or lymphoid	29, 7.57%
Musculoskeletal	24, 6.27%
Other ^a^	65, 16.97%
U.S. Region (n, %)	
Midwest	61, 15.93%
Northeast	86, 22.45%
South	152, 39.69%
Southeast	65, 16.97%
West	19, 4.96%
Visits attended (Mean ± SD)	14.23 ± 12.37
Weeks attended (Mean ± SD)	11.17 ± 11.25
Achieved the MCID on PRO (n, %) ^b^	179, 46.7%

Note: PRO = patient reported outcome. ^a^ Other includes: brain, eye, central nervous system, colorectal, endocrine/neuroendocrine, gastrointestinal, genitourinary, gynecologic, head and neck, lung and skin. ^b^ PRO were selected by the treating therapist and include: the Quick Disabilities of the Arm, Shoulder, and Hand (MCID = 15.91 points [28]), the Lower Extremity Functional Scale (MCID = 9 points [29]), and the Modified Low Back Pain Disability Questionnaire (MCID = 7.5 points) [30].

## Data Availability

Data available upon request.

## Supplementary Materials

The following supporting information can be downloaded at: https://www.mdpi.com/article/10.3390/healthcare11030348/s1, Supplementary Table S1, Individual and rehabilitation characteristics of patients who attended outpatient cancer rehabilitation and completed Net Promoter Survey^®^ (NPS) vs. those who did not complete NPS.

## References

[1] Berwick D.M., Nolan T.W., Whittington J. (2008). The Triple Aim: Care, health, and cost. Health Aff..

[2] Oben P. (2020). Understanding the Patient Experience: A Conceptual Framework. J. Patient Exp..

[3] Pergolotti M., Deal A.M., Lavery J., Reeve B.B., Muss H.B. (2015). The prevalence of potentially modifiable functional deficits and the subsequent use of occupational and physical therapy by older adults with cancer. J. Geriatr. Oncol..

[4] Schmidt M.E., Wiskemann J., Steindorf K. (2018). Quality of life, problems, and needs of disease-free breast cancer survivors 5 years after diagnosis. Qual. Life Res..

[5] Mazariego C.G., Juraskova I., Campbell R., Smith D.P. (2020). Long-term unmet supportive care needs of prostate cancer survivors: 15-year follow-up from the NSW Prostate Cancer Care and Outcomes Study. Support. Care Cancer.

[6] Weaver R., O’Connor M., Sobhi S., Smith R.C., Halkett G. (2020). The unmet needs of patients with sarcoma. Psychooncology.

[7] Sleight A., Gerber L.H., Marshall T.F., Livinski A., Alfano C.M., Harrington S., Flores A.M., Virani A., Hu X., Mitchell S.A. (2022). Systematic Review of Functional Outcomes in Cancer Rehabilitation. Arch. Phys. Med. Rehabil..

[8] Hunter E.G., Gibson R.W., Arbesman M., D’Amico M. (2017). Systematic Review of Occupational Therapy and Adult Cancer Rehabilitation: Part 2. Impact of Multidisciplinary Rehabilitation and Psychosocial, Sexuality, and Return-to-Work Interventions. Am. J. Occup. Ther..

[9] Pergolotti M., Covington K.R.M., Lightner A.N.D., Bertram J.D., Thess M.P., Sharp J., Spraker M.M., Williams G.R., Manning P.M. (2020). Association of Outpatient Cancer Rehabilitation with Patient-Reported Outcomes and Performance-Based Measures of Function. Rehabil. Oncol..

[10] Wood K.C., Bertram J., Kendig T., Hidde M., Leiser A., de Meritens A.B., Pergolotti M. (2022). Community-based outpatient cancer rehabilitation services for women with gynecologic cancer: Acceptability and impact on patient-reported outcomes. Support. Care Cancer.

[11] Wood K.C., Hidde M., Kendig T., Pergolotti M. (2022). Community-based outpatient rehabilitation for the treatment of breast cancer-related upper extremity disability: An evaluation of practice-based evidence. Breast Cancer.

[12] Stout N.L., Mina D.S., Lyons K.D., Robb K., Silver J.K. (2020). A systematic review of rehabilitation and exercise recommendations in oncology guidelines. CA A Cancer J. Clin..

[13] Alderman G., Semple S., Cesnik R., Toohey K. (2020). Health Care Professionals’ Knowledge and Attitudes Toward Physical Activity in Cancer Patients: A Systematic Review. Semin. Oncol. Nurs..

[14] Pergolotti M., Alfano C.M., Cernich A.N., Yabroff K.R., Manning P.R., Moor J.S., Hahn E.E., Cheville A.L., Mohile S.G. (2019). A health services research agenda to fully integrate cancer rehabilitation into oncology care. Cancer.

[15] Nadler M., Bainbridge D., Tomasone J., Cheifetz O., Juergens R.A., Sussman J. (2017). Oncology care provider perspectives on exercise promotion in people with cancer: An examination of knowledge, practices, barriers, and facilitators. Support. Care Cancer.

[16] Brick R., Lyons K.D., Bender C., Eilers R., Ferguson R., Pergolotti M., Toto P., Skidmore E., Leland N.E. (2021). Factors influencing utilization of cancer rehabilitation services among older breast cancer survivors in the USA: A qualitative study. Support. Care Cancer.

[17] Nadler M.B., Rose A.A., Prince R., Eng L., Lott A., Grant R.C., Jones J.M., Enright K. (2021). Increasing Referrals of Patients with Gastrointestinal Cancer to a Cancer Rehabilitation Program: A Quality Improvement Initiative. JCO Oncol. Pract..

[18] Pergolotti M., Bailliard A., McCarthy L., Farley E., Covington K.R., Doll K.M. (2020). Women’s Experiences after Ovarian Cancer Surgery: Distress, Uncertainty, and the Need for Occupational Therapy. Am. J. Occup. Ther..

[19] Dennett A.M., Peiris C.L., Taylor N.F., Reed M.S., Shields N. (2018). ‘A good stepping stone to normality’: A qualitative study of cancer survivors’ experiences of an exercise-based rehabilitation program. Support. Care Cancer.

[20] Nielsen S., Ringborg C.H., Schandl A., Lagergren P. (2021). A qualitative study exploring patient’s experiences of oesophageal cancer surgery, through their personal advice to future patients. Eur. J. Oncol. Nurs..

[21] Adams C., Walpola R., Schembri A.M., Harrison R. (2022). The ultimate question? Evaluating the use of Net Promoter Score in healthcare: A systematic review. Health Expect..

[22] Lehmann J., Rothmund M., Riedl D., Rumpold G., Grote V., Fischer M.J., Holzner B. (2021). Clinical Outcome Assessment in Cancer Rehabilitation and the Central Role of Patient-Reported Outcomes. Cancers.

[23] Motheral B., Brooks J., Clark M.A., Crown W.H., Davey P., Hutchins D., Martin B.C., Stang P. (2003). A Checklist for Retrospective Database Studies—Report of the ISPOR Task Force on Retrospective Databases. Value Health.

[24] Lis C.G., Rodeghier M., Gupta D. (2011). The relationship between perceived service quality and patient willingness to recommend at a national oncology hospital network. BMC Health Serv. Res..

[25] Klepps R. (2018). NPS^®^ in Health Care: Leveraging Loyal Patients to Drive New Business and Improve Revenue. https://www.webpt.com/blog/nps-in-health-care-leveraging-loyal-patients-to-drive-new-business-and-improve-revenue/.

[26] Bain & Company Measuring Your Net Promoter Score. https://www.netpromotersystem.com/about/measuring-your-net-promoter-score.

[27] Copay A.G., Subach B.R., Glassman S.D., Polly D.W., Schuler T.C. (2007). Understanding the minimum clinically important difference: A review of concepts and methods. Spine J..

[28] Franchignoni F., Vercelli S., Giordano A., Sartorio F., Bravini E., Ferriero G. (2014). Minimal Clinically Important Difference of the Disabilities of the Arm, Shoulder and Hand Outcome Measure (DASH) and Its Shortened Version (QuickDASH). J. Orthop. Sports Phys. Ther..

[29] Binkley J.M., Stratford P., Lott S.A., Riddle D.L. (1999). The Lower Extremity Functional Scale (LEFS): Scale Development, Measurement Properties, and Clinical Application. Phys. Ther..

[30] Fritz J.M., Irrgang J.J. (2001). A Comparison of a Modified Oswestry Low Back Pain Disability Questionnaire and the Quebec Back Pain Disability Scale. Phys. Ther..

[31] Teddlie C., Tashakkori A. (2008). Foundations of Mixed Methods Research: Integrating Quantitative and Qualitative Approaches in the Social and Behavioral Sciences.

[32] Vaismoradi M., Jones J., Turunen H., Snelgrove S. (2015). Theme development in qualitative content analysis and thematic analysis. J. Nurs. Educ. Pract..

[33] Graneheim U.H., Lundman B. (2004). Qualitative content analysis in nursing research: Concepts, procedures and measures to achieve trustworthiness. Nurse Educ. Today.

[34] Cunningham M., Wells M. (2017). Qualitative analysis of 6961 free-text comments from the first National Cancer Patient Experience Survey in Scotland. BMJ Open.

[35] St. Pierre E.A., Jackson A.Y. (2014). Qualitative Data Analysis after Coding. Qual. Inq..

[36] Wilberforce M., Poll S., Langham H., Worden A., Challis D. (2018). Measuring the patient experience in community mental health services for older people: A study of the Net Promoter Score using the Friends and Family Test in England. Int. J. Geriatr. Psychiatry.

[37] Sizmur S., Graham C., Walsh J. (2014). Influence of patients’ age and sex and the mode of administration on results from the NHS Friends and Family Test of patient experience. J. Health Serv. Res. Policy.

[38] Hamilton D.F., Lane J.V., Gaston P., Patton J.T., Macdonald D.J., Simpson A.H.R.W., Howie C.R. (2014). Assessing treatment outcomes using a single question: The Net Promoter Score. Bone Jt. J..

[39] Beresford L., Norwood T. (2022). The Effect of Mobile Care Delivery on Clinically Meaningful Outcomes, Satisfaction, and Engagement Among Physical Therapy Patients: Observational Retrospective Study. JMIR Rehabil. Assist. Technol..

[40] Hong M., Loeb J., Yang M., Bailey J.F. (2022). Postoperative Outcomes of a Digital Rehabilitation Program after Total Knee Arthroplasty: Retrospective, Observational Feasibility Study. JMIR Form. Res..

[41] Bettger J.P., Green C.L., Holmes D.N., Chokshi A., Mather R.C., Hoch B.T., de Leon A.J., Aluisio F., Seyler T.M., Del Gaizo D.J. (2020). Effects of Virtual Exercise Rehabilitation In-Home Therapy Compared with Traditional Care after Total Knee Arthroplasty: VERITAS, a Randomized Controlled Trial. J. Bone Jt. Surg..

[42] Kohli R., Gupta A. (2022). A Cross-Sectional Study to Assess Quality of Care and Patient Satisfaction Using Theranow Telerehabilitation Program Post-THR and TKR Surgeries. J. Sci. Res. Med. Biol. Sci..

[43] Dietz J.H. (1969). Rehabilitation of the cancer patient. Med. Clin. North Am..

