# Supplementary material for: Understanding Patient Experience with Outpatient Cancer Rehabilitation Care
Source: Healthcare (Basel). 2023 Jan 25;11(3):348. doi: 10.3390/healthcare11030348 (PMC9914453; doi:10.3390/healthcare11030348)
Supplement: Supplementary file 1 [file healthcare-11-00348-s001.zip › healthcare-2126062-supplementary.pdf]

Supplement Table S1. Individual and rehabilitation characteristics of patients who attended outpatient cancer rehabilitation and completed Net Promoter Survey® (NPS) vs. those who did not complete NPS

	**NPS available (n=383)**	**NPS unavailable (n=1,018)**	**Between-group comparison, p-value**
Age (Mean ± SD)	60.51 ± 12.02	60.55 ± 13.46	*t* [1399] = 0.05, *p* = 0.96
Sex (N, %)			
Female	323, 84.33%	843, 82.81%	χ2 [1] = 0.46, *p* = 0.50
Male	60, 15.67%	175, 17.19%	
Cancer type (N, %)			
Breast	265, 69.19%	623, 61.2%	χ2 [3] = 7.80, *p* = 0.05
Heme or lymphoid	29, 7.57%	98, 9.63%	
Musculoskeletal	24, 6.27%	74, 7.27%	
Other	65, 16.97%	223, 21.91%	
Rehabilitation discipline (N, %)			
PT	341, 89.03%	865, 84.97%	χ2 [1] = 3.84, *p* = 0.05
OT	42, 10.97%	153, 15.03%	
Insurance type (N, %)			
Federally funded insurance	196, 51.17%	517, 50.79%	χ2 [2] = 0.36, *p* = 0.83
Private insurance	179, 46.74%	474, 46.56%	
Other	8, 2.09%	27, 2.65%	
Visits attended (Mean ± SD)	14.23 ± 12.37	14.71 ± 14.7	*t* [1399] = 0.57, *p* = 0.57
Weeks attended (Mean ± SD)	11.17 ± 11.25	11.99 ± 11.41	*t* [1399] = 1.28, *p* = 0.20
Achieved the MCID on PRO (N, %) [b]	179, 46.74%	481, 47.25%	χ2 [1] = 0.03, p = 0.86

*Between-groups difference, *p*<.05

**Other includes: Brain, Eye, CNS, Colorectal, endocrine and neuroendocrine, gastrointestinal, genitourinary, gynecologic, head and neck, lung and respiratory and skin.
